# TRIM35 mediates protection against influenza infection by activating TRAF3 and degrading viral PB2

**DOI:** 10.1007/s13238-020-00734-6

**Published:** 2020-06-19

**Authors:** Nan Sun, Li Jiang, Miaomiao Ye, Yihan Wang, Guangwen Wang, Xiaopeng Wan, Yuhui Zhao, Xia Wen, Libin Liang, Shujie Ma, Liling Liu, Zhigao Bu, Hualan Chen, Chengjun Li

**Affiliations:** State Key Laboratory of Veterinary Biotechnology, Harbin Veterinary Research Institute, Chinese Academy of Agricultural Sciences, Harbin, 150069 China

**Keywords:** influenza A virus, PB2, TRIM35, TRAF3, ubiquitination, antiviral immunity

## Abstract

Tripartite motif (TRIM) family proteins are important effectors of innate immunity against viral infections. Here we identified TRIM35 as a regulator of TRAF3 activation. Deficiency in or inhibition of TRIM35 suppressed the production of type I interferon (IFN) in response to viral infection. *Trim35*-deficient mice were more susceptible to influenza A virus (IAV) infection than were wild-type mice. TRIM35 promoted the RIG-I-mediated signaling by catalyzing Lys63-linked polyubiquitination of TRAF3 and the subsequent formation of a signaling complex with VISA and TBK1. IAV PB2 polymerase countered the innate antiviral immune response by impeding the Lys63-linked polyubiquitination and activation of TRAF3. TRIM35 mediated Lys48-linked polyubiquitination and proteasomal degradation of IAV PB2, thereby antagonizing its suppression of TRAF3 activation. Our *in vitro* and *in vivo* findings thus reveal novel roles of TRIM35, through catalyzing Lys63- or Lys48-linked polyubiquitination, in RIG-I antiviral immunity and mechanism of defense against IAV infection.

**Electronic supplementary material:**

The online version of this article (10.1007/s13238-020-00734-6) contains supplementary material, which is available to authorized users.

## Introduction

Influenza A virus (IAV) is a human respiratory pathogen that causes seasonal epidemics and occasional global pandemics associated with significant morbidity and mortality. The genome of IAV consists of eight single-stranded negative-sense RNA segments, encoding ten essential proteins and up to eight accessary proteins (Yamayoshi et al., 2016). The viral polymerase basic protein 2 (PB2) plays an important role in the adaptation and pathogenesis of avian influenza virus in mammalian hosts (Subbarao et al., 1993; Hatta et al., 2001; Gabriel et al., 2005; Li et al., 2005; Min et al., 2013). It possesses two nuclear localization signals that enable it to accumulate in the nucleus of infected cells (Mukaigawa and Nayak, 1991), where it forms the polymerase complex with polymerase basic protein 1 (PB1) and polymerase acidic protein (PA) to catalyze the transcription and replication of the viral genome. Specifically, the PB2 cap-binding domain, together with the PA endonuclease, functions in cap-snatching to generate 5′-capped primers that are required to initiate viral RNA transcription (Plotch et al., 1981; Dias et al., 2009; Yuan et al., 2009).

Innate immunity plays a crucial role in host defense against invading pathogens. To defend against IAV infection, RIG-I (retinoic acid-inducible gene I) acts as a dominant innate immune sensor in the cytoplasm, where it recognizes and binds to the 5′ppp-dsRNA panhandle structure that is formed by the conserved 5′ and 3′ ends of the viral RNAs (Baum et al., 2010; Weber et al., 2013). Upon binding the viral RNA through the central helicase domain, RIG-I undergoes a conformational change, thereby releasing the N-terminal caspase activation and recruitment domains (CARDs) from the repression of the C-terminal repressor domain (RD) (Kowalinski et al., 2011). Subsequently, through a CARD–CARD interaction, activated RIG-I interacts with the virus-induced signaling adapter (VISA) (also known as MAVS, Cardif, or IPS-1; hereinafter, VISA is used) (Kawai et al., 2005; Meylan et al., 2005; Seth et al., 2005; Xu et al., 2005). This, via TNF receptor-associated factor 3 (TRAF3) (Saha et al., 2006), leads to the recruitment of TANK-binding kinase 1 (TBK1) and IkB kinase-ε (IKKε) to activate interferon regulatory factor 3 (IRF3) (Fitzgerald et al., 2003; Hacker et al., 2011). Activated IRF3 then translocates from the cytoplasm to the nucleus and binds to interferon stimulated response elements (ISRE) in the promoter of IFN-β and IFN-α, resulting in the production of type I IFNs (Loo and Gale, 2011; Yoneyama et al., 2015).

Tripartite motif (TRIM) proteins in humans constitute a large family of at least 80 members, which share three conserved domains: an N-terminal Really Interesting New Gene (RING) domain, one or two B-Boxes (B1/B2), and a coiled coil (CC) domain (RBCC) (van Gent et al., 2018). The most prominent role of TRIM proteins is to function in innate immunity and antiviral responses (Ozato et al., 2008; Rajsbaum et al., 2014). The RING domains in TRIM proteins enable them to function as ubiquitin E3 ligases that are actively involved in the ubiquitination of target proteins (Joazeiro and Weissman, 2000), mostly through Lys48 (K48)- or Lys63 (K63)-linkages (Davis and Gack, 2015). The role of TRIM proteins in innate signaling pathways essentially relies on their ability to catalyze ubiquitination.

Here, we identified TRIM35 (tripartite motif containing 35, also known as HLS5, MAIR) as a positive regulator of the RIG-I-mediated innate immune pathway upon infection with IAV, SeV, or VSV. We found that TRIM35 interacted with TRAF3 and promoted its K63-linked ubiquitination. We also found that the PB2 protein of IAV impeded K63-linked ubiquitination of TRAF3 and disrupted the formation of the VISA–TRAF3 complex. TRIM35 interacted with IAV PB2 and degraded PB2 through K48-linked ubiquitination, thereby antagonizing the PB2-induced impairment of K63-linked ubiquitination and activation of TRAF3. Our study thus revealed an important mechanism by which TRIM35 mediates innate immunity and antiviral activity against IAV infection.

## Results

### TRIM35 positively regulates RIG-I-mediated IFN-β signaling

**Figure 1:**
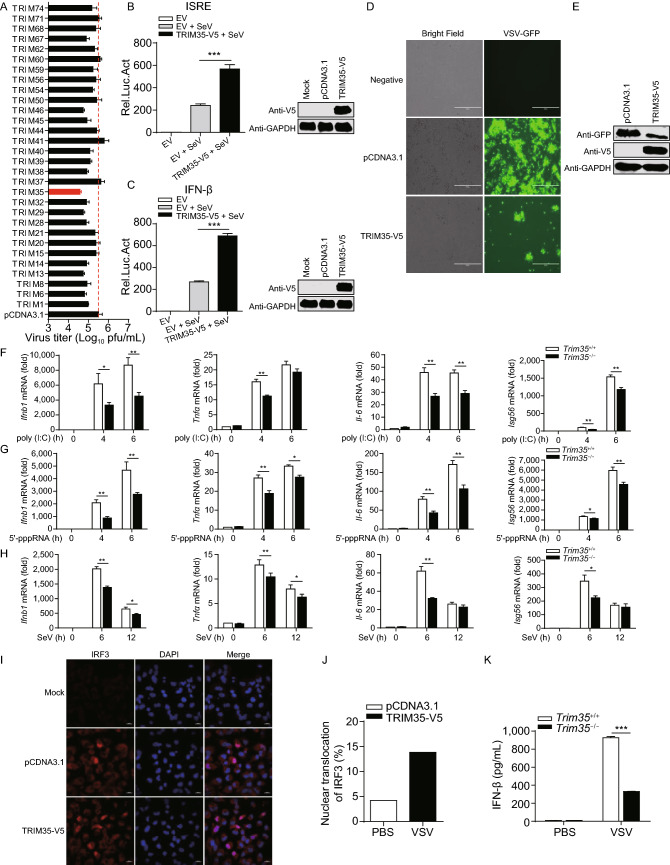
**Identification of TRIM35 as a positive regulator of RIG-I-mediated IFN-β signaling**. (A) Virus yield in HEK293 cells transfected for 24 h to express one of the 30 TRIM proteins, followed by infection with WSN (H1N1) virus (MOI = 0.1). Supernatants were collected at 48 h p.i. and subjected to plaque assay on MDCK cells. (B and C) ISRE (B) or IFN-β (C) promoter luciferase reporter assay of HEK293T cells transfected for 24 h with TRIM35-V5 expression plasmid or empty vector (EV), followed by stimulation with SeV for 12 h. Results are expressed relative to *Renilla* luciferase activity. (D and E) Replication of VSV-GFP virus. HEK293T cells were transfected with plasmid expressing TRIM35-V5 or control vector for 24 h, and then infected with SeV for 12 h. Supernatants were inactivated with ultraviolet (UV) light. Fresh HEK293T cells were then incubated with the UV-inactivated supernatants for 24 h, followed by infection with VSV-GFP for 12 h. VSV-GFP replication was analyzed by fluorescence microscopy (D) and immunoblot (IB) analysis (E). Scale bars, 100 µm. (F–H) qRT-PCR analysis of *Ifnb1*, *Tnfα*, *Il6* or *Isg56* mRNA in *Trim35*^+/+^ and *Trim35*^−/−^ peritoneal macrophages transfected with poly(I:C) (F) or 5′-pppRNA (G) or infected with SeV (H). (I) IFA of IRF3 protein expression. HEK293T cells were transfected for 36 h with plasmid expressing TRIM35-V5 or control vector. Cells were then left untreated or stimulated with 5′-pppRNA for 8 h, followed by immunostaining of IRF3 and fluorescence microscopy. (J) Number of cells showing nuclear localization of IRF3 from (I). Results shown are calculated from at least three hundred cells. (K) ELISA of serum IFN-β concentrations. Six-week-old female *Trim35*^+/+^ and *Trim35*^−/−^ mice (five per group) were infected with VSV (2 × 10^7^ pfu/mouse) by intravenous injection, and serum IFN-β concentrations were measured on day 5 p.i. Data are representative of at least three independent experiments. Means ± SD are shown in (A–C, F–H) (*n* = 3), and (K) (*n* = 5). Two-tailed unpaired *t*-test was used for the statistical analysis, *P < 0.05, **P < 0.01, ***P < 0.001

To systematically investigate human TRIM proteins with antiviral activity against IAV, we performed a preliminary viral replication screen with 30 TRIM proteins transiently expressed in HEK293 cells. Among these 30 TRIM proteins, we identified TRIM35 as a potent inhibitory effector of IAV replication. Upregulation of TRIM35 expression in HEK293 cells led to an approximately 10-fold reduction in virus titer at 48 h post-infection (p.i.) (Fig. 1A).

Given the importance of TRIM proteins in mediating antiviral innate immunity, we examined the role of TRIM35 in innate immune signaling. To this end, HEK293T cells were co-transfected with a TRIM35 expression construct and an ISRE- or IFN-β-luciferase reporter, and then infected with Sendai virus (SeV). The luciferase assay showed that overexpression of TRIM35 markedly enhanced SeV-triggered activation of the ISRE and IFN-β promoter (Fig. 1B and 1C). We next used GFP-expressing vesicular stomatitis virus (VSV-GFP) to infect HEK293T cells that expressed exogenous TRIM35. Fluorescence microscopy images showed that overexpression of TRIM35 substantially inhibited the viral replication relative to that of the control vector-transfected cells (Fig. 1D). The inhibitory effect of TRIM35 overexpression on the replication of VSV-GFP was confirmed by the reduced level of GFP expression as determined by immunoblot analysis (Fig. 1E).

To investigate the biological role of TRIM35, we generated *Trim35*^−/−^ C57BL/6J mice by use of CRISPR/Cas9-mediated gene targeting. The targeting construct was designed to delete exons 4–6 of the mouse *Trim35* gene (Fig. S1A). Deletion of *Trim35* was confirmed by sequencing, and knockout of the TRIM35 protein was confirmed by immunoblot analysis of lung extracts from *Trim35*^−/−^ mice (Fig. S1B). *Trim35*^−/−^ mice are viable, normal in size, and do not display any gross physical or behavioral abnormalities (data not shown). We prepared peritoneal macrophages from *Trim35*^+/+^ and *Trim35*^−/−^ mice, and then stimulated them with RIG-I receptor ligands such as poly(I:C), RNA mimics (5′-pppRNA), or SeV. We observed that the expression of *Ifnb1* after stimulation with poly(I:C) or 5′-pppRNA or infection with SeV was significantly lower in *Trim35*^−/−^ macrophages than in *Trim35*^+/+^ macrophages (Fig. 1F–H). The expression of *Tnfα*, *Il6*, and *Isg56*, which are downstream genes in RIG-I signaling, was also lower in *Trim35*^−/−^ macrophages than their *Trim35*^+/+^ counterparts (Fig. 1F–H).

We also established a stable TRIM35-overexpressing THP-1 cell line by using the retroviral-mediated system (Fig. S2A). We found that overexpression of TRIM35 in THP-1 cells significantly enhanced transcription of *IFNB1*, *TNFα*, *IL6*, and *ISG56* after infection with SeV (Fig. S2B). In contrast, siRNA-mediated knockdown of *Trim35* transcription in RAW264.7 cells (Fig. S2C) reduced SeV-induced expression of *Ifnb1*, *Tnfα*, *Il6*, and *Isg56* (Fig. S2D).

IRF3, which dimerizes in the cytoplasm upon activation by phosphorylated TBK1, translocates to the nucleus where it binds the IFN-β promoter, resulting in the induction of IFN-β (Loo and Gale, 2011; Yoneyama et al., 2015). To determine the effect of TRIM35 on IRF3 activation, we examined IRF3 localization in TRIM35-overexpressing and control HEK293T cells stimulated by 5′-pppRNA. Fluorescence microscopy images showed that overexpression of TRIM35 significantly enhanced the number of cells exhibiting nuclear localization of IRF3 at 8 h p.i. (Fig. 1I and 1J).

To further determine the effect of TRIM35 knockout on the production of IFN-β *in vivo*, we infected *Trim35*^+/+^ and *Trim35*^−/−^ mice with VSV and determined the serum levels of IFN-β on day 5 p.i. by use of an ELISA. We found that VSV-induced IFN-β production was significantly decreased in the serum of *Trim35*^−/−^ mice compared with that of *Trim35*^+/+^ mice (Fig. 1K).

Together, these data demonstrate that TRIM35 positively regulates IFN-β signaling and antiviral responses mediated by RIG-I.

### TRIM35 is induced by RNA virus infection and IFN-β treatment

**Figure 2:**
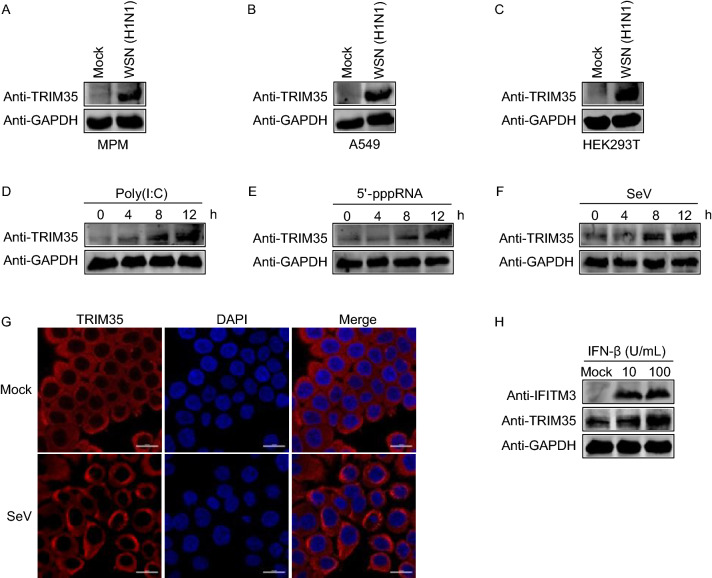
**TRIM35 expression is induced by viral infection and IFN-β treatment**. (A–C) IB analysis of TRIM35 expression in mouse peritoneal macrophages (A), A549 (B), or HEK293T (C) cells infected with WSN (H1N1) virus (MOI = 3) for 20 h. MPM, mouse peritoneal macrophages. (D–F) IB analysis of TRIM35 expression in mouse peritoneal macrophages stimulated with poly(I:C) (D) or 5′-pppRNA (E) or were infected with SeV (F) at the indicated timepoints. (G) IFA for TRIM35 expression in Hela cells that were mock infected or infected with SeV for 8 h. Scale bars, 20 μm. (H) IB analysis of TRIM35 expression in mouse peritoneal macrophages treated with the indicated amounts of IFN-β for 12 h. The interferon-induced transmembrane protein 3 (IFITM3) was included as a positive control. Data are representative of at least three independent experiments

To assess how TRIM35 responds to IAV infection, we examined the expression of TRIM35 in IAV-infected mouse peritoneal macrophages. The expression of TRIM35 was dramatically induced after A/WSN/33 (WSN, H1N1) infection compared with that in uninfected control cells (Fig. 2A). In agreement with our observations in mouse peritoneal macrophages, we observed that infection with IAV greatly increased the expression of TRIM35 in A549 and HEK293T cells (Fig. 2B and 2C). Similarly, expression of the TRIM35 protein was enhanced in mouse peritoneal macrophages after stimulation with poly(I:C) and 5′-pppRNA (Fig. 2D and 2E). Indeed, increasing the duration of stimulation induced the expression of more TRIM35 protein (Fig. 2D and 2E). In addition, the level of TRIM35 increased over time after SeV infection in mouse peritoneal macrophages (Fig. 2F). We also visualized the localization of TRIM35 in SeV-infected Hela cells, and found that the expression of TRIM35 also increased (Fig. 2G).

Since IAV is intrinsically sensitive to the antiviral activity of type I IFN and TRIM35 is a factor in restricting the replication of IAV, we speculated that type I IFN might regulate TRIM35 expression. Therefore, we treated mouse peritoneal macrophages with IFN-β and determined the expression of TRIM35 by immunoblot analysis. IFN-β treatment led to increased expression of TRIM35 in a dose-dependent manner (Fig. 2H), indicating that TRIM35 is an antiviral interferon-stimulated gene (ISG) induced by type I IFN.

### TRIM35 suppresses IAV replication *in vitro* and *in vivo*

**Figure 3:**
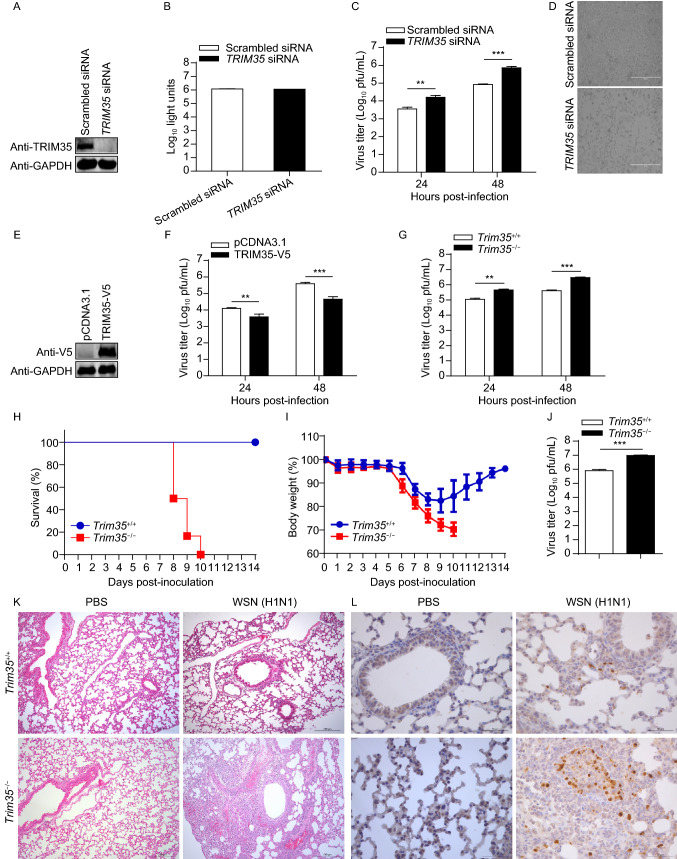
**TRIM35 dampens the replication and virulence of IAV**. (A) IB analysis of TRIM35 expression in A549 cells transfected with siRNA targeting *TRIM35* or with scrambled siRNA for 48 h. (B) Cell viability of siRNA-treated A549 cells as in (A). (C and D) Replication of WSN (H1N1) virus (MOI = 0.1) in siRNA-treated A549 cells as in (A). Supernatants collected at the indicated timepoints were subjected to plaque assay on MDCK cells (C). Virus infected cells were visualized by bright-field microscopy (D). Scale bars, 400 µm. (E) IB analysis of TRIM35 expression in A549 cells transfected with plasmid expressing TRIM35-V5 or control vector. (F) Replication of WSN (H1N1) virus (MOI = 0.1) in TRIM35-overexpressing or control A549 cells as in (E). Supernatants collected at the indicated timepoints were subjected to plaque assay on MDCK cells. (G) Replication of WSN (H1N1) virus (MOI = 0.1) in peritoneal macrophages isolated from *Trim35*^+/+^ and *Trim35*^−/−^ mice. Supernatants collected at the indicated timepoints were subjected to plaque assay on MDCK cells. (H and I) Survival (H) and body weight (I) of 6-week-old female *Trim35*^+/+^ and *Trim35*^−/−^ mice (*n* = 6 per group) intranasally infected with WSN (H1N1) virus (2 × 10^3^ pfu/mouse). (J) Titers of WSN (H1N1) virus, determined by plaque assay, in the lungs of *Trim35*^+/+^ and *Trim35*^−/−^ mice on day 3 p.i. as in (H and I). (K and L) Hematoxylin-and-eosin staining (K) or immunohistochemical (IHC) staining (L) of lung sections prepared on day 3 p.i. from *Trim35*^+/+^ and *Trim35*^−/−^ mice as in (H and I). Scale bars, 200 µm (K) and 50 µm (L). Data are representative of at least three independent experiments. Means ± SD are shown in (B, C, F, G) (*n* = 3), and (J) (*n* = 6). The values for body weights in (I) are means ± SD from live mice. Two-tailed unpaired *t*-test was used for the statistical analysis, **P < 0.01, ***P < 0.001

To explore the role of TRIM35 in IAV infection, A549 cells were transfected with siRNA targeting *TRIM35* and then infected with WSN (H1N1) virus. *TRIM35*-specific siRNA treatment efficiently reduced the expression of TRIM35 compared with scrambled siRNA without affecting cell viability (Fig. 3A and 3B). SiRNA-mediated knockdown of TRIM35 expression increased production of infectious virus by 10-fold (Fig. 3C); more severe cytopathic effect induced by WSN (H1N1) virus infection was observed in cells that were treated with *TRIM35*-specific siRNA compared with scrambled siRNA (Fig. 3D). Consistent with these data, overexpression of TRIM35 in A549 cells dramatically inhibited the replication of WSN (H1N1) virus (Fig. 3E and 3F). Furthermore, we found that the growth titers of WSN (H1N1) virus were significantly greater in primary peritoneal macrophages of *Trim35*^−/−^ mice than those derived from *Trim35*^+/+^ mice (Fig. 3G).

To evaluate the importance of TRIM35 in mediating the host defense against viral infection *in vivo*, *Trim35*^+/+^ and *Trim35*^−/−^ mice were intranasally infected with 2 × 10^3^ PFU of WSN (H1N1) influenza virus, and their survival and body weight changes were monitored daily for 14 days. We found that all six *Trim35*^−/−^ mice died of their infection on day 8, 9, or 10 (Fig. 3H). In contrast, 100% of *Trim35*^+/+^ mice survived (Fig. 3H). In addition, *Trim35*^−/−^ mice continued to lose body weight until death after infection, whereas all of the *Trim35*^+/+^ mice gained body weight at the late stage of infection (Fig. 3I).We also measured virus titers in lung homogenates on day 3 p.i. and found that the viral load was dramatically increased in *Trim35*^−/−^ mice compared with that in *Trim35*^+/+^ mice (Fig. 3J). Histopathology examination revealed significantly more severe bronchopneumonia with more prominent viral antigen expression in the lungs of *Trim35*^−/−^ mice compared with *Trim35*^+/+^ mice after viral infection (Fig. 3K and 3L). These data indicate that TRIM35 suppresses virus replication and alleviates virus-induced pathogenicity during IAV infection *in vivo*.

### TRIM35 targets TRAF3

**Figure 4:**
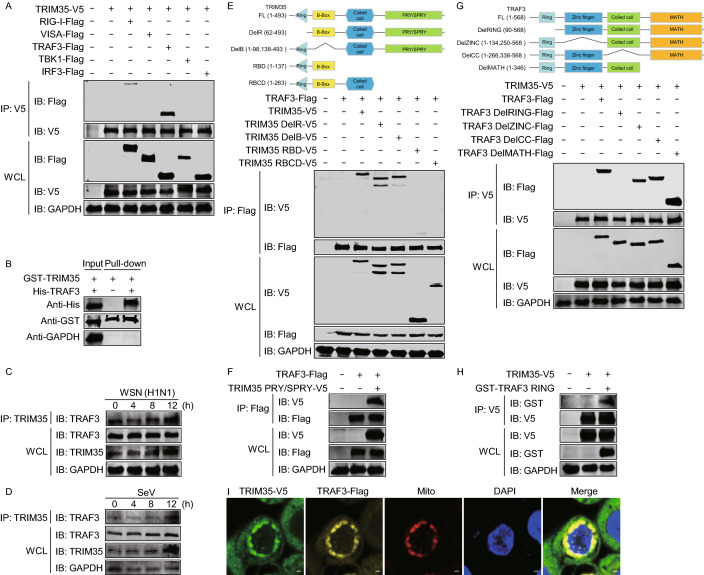
**TRIM35 interacts with TRAF3**. (A) Co-immunoprecipitation (co-IP) and IB analysis of HEK293T cells expressing TRIM35-V5 along with Flag-tagged RIG-I, VISA, TRAF3, TBK1, or IRF3. WCL, IB analysis of whole cell lysates without IP. (B) GST pull-down assay of purified His-tagged TRAF3 and lysates of HEK293T cells expressing GST-TRIM35. (C and D) Co-IP and IB analysis of the interaction between TRIM35 and TRAF3 in mouse peritoneal macrophages infected for 0–12 h with WSN (H1N1) virus (C) or SeV (D). (E) Binding of V5-tagged TRIM35 or its truncation mutants with TRAF3-Flag in transiently transfected HEK293T cells, as determined by co-IP and IB analysis. Human TRIM35 domains and truncation mutants were shown on top. (F) Binding of the V5-tagged TRIM35 PRY/SPRY domain with TRAF3-Flag in transiently transfected HEK293T cells, as determined by co-IP and IB analysis. (G) Binding of Flag-tagged TRAF3 or its truncation mutants with TRIM35-V5 in transiently transfected HEK293T cells, as determined by co-IP and IB analysis. Human TRAF3 domains and truncation mutants were shown on top. (H) Binding of the GST-tagged TRAF3 RING domain with TRIM35-V5 in transiently transfected HEK293T cells, as determined by co-IP and IB analysis. (I) Co-localization of TRIM35 and TRAF3 proteins in HEK293T cells expressing TRIM35-V5 and TRAF3-Flag for 36 h. Mitochondria and nuclei were stained using MitoTracker Red and DAPI, respectively. Data are representative of at least three independent experiments

RIG-I-mediated innate immune signaling is critical for the host defense against RNA virus infection (Kawai and Akira, 2006; Thompson et al., 2011). To identify the molecules in the RIG-I signaling pathway that are regulated by TRIM35, we performed co-immunoprecipitation (co-IP) experiments in HEK293T cells to examine the interaction between TRIM35 and the key adaptors of the RIG-I pathway. TRIM35 associated clearly with TRAF3, only weakly with RIG-I, but not with VISA, TBK1, or IRF3 (Fig. 4A). The TRIM35-TRAF3 interaction was confirmed in a GST pull-down assay. His-tagged TRAF3 was only pulled down in the presence of GST-tagged TRIM35 (Fig. 4B). We performed an additional co-IP experiment in mouse peritoneal macrophages that were infected with IAV or SeV. More TRAF3 protein was immunoprecipitated by the gradually increasing levels of TRIM35 expression across the infection course (Fig. 4C and 4D), indicating TRIM35 interacted with TRAF3 in vivo during natural viral infection. TRAF3 serves as a link between the adaptor VISA and downstream regulatory kinases that are essential for IRF3 activation. These data suggest that TRIM35 exerts its role in activating antiviral innate immunity via a strong interaction with TRAF3.

TRIM35 is composed of a RING finger domain (residues 21–61), a B-box domain (residues 96–137), a coiled-coil region domain (residues 145–263), and a PRY/SPRY domain (residues 301–484) (Chen et al., 2015). To determine which domain of TRIM35 is essential for its interaction with TRAF3, we constructed several TRIM35 deletion mutants and assessed them in co-IP experiments (Fig. 4E). We found that deletion of the C-terminal PRY/SPRY domain of TRIM35 led to the loss of the interaction with TRAF3. Additionally, the PRY/SPRY domain of TRIM35 was sufficient for the interaction with TRAF3 (Fig. 4F).

TRAF3 contains four structural and functional domains: a RING finger domain (residues 68–77), a zinc finger domain (residues 135–249), a coiled coil domain (residues 267–338), and a MATH domain (residues 415–560) (Zhu et al., 2015). To map the TRAF3 domains involved in TRIM35 binding, we generated four TRAF3 deletion mutants, each missing an individual domain (Fig. 4G). Co-IP assays in HEK293T cells indicated that deletion of the TRAF3-RING domain abolished the interaction with TRIM35 (Fig. 4G), and the TRAF3-RING domain alone was sufficient for the association with TRIM35 (Fig. 4H).

On the basis of these findings, we then asked whether TRAF3 and TRIM35 co-localize in cells. Confocal microscopy revealed that these two proteins co-localized and associated with mitochondria (Fig. 4I). Mitochondria have emerged as important platforms for intracellular innate immune signaling. The co-localization of these two proteins in mitochondria implies the importance of TRIM35 in antiviral innate immunity.

### TRIM35 promotes the K63-linked polyubiquitination of TRAF3

**Figure 5:**
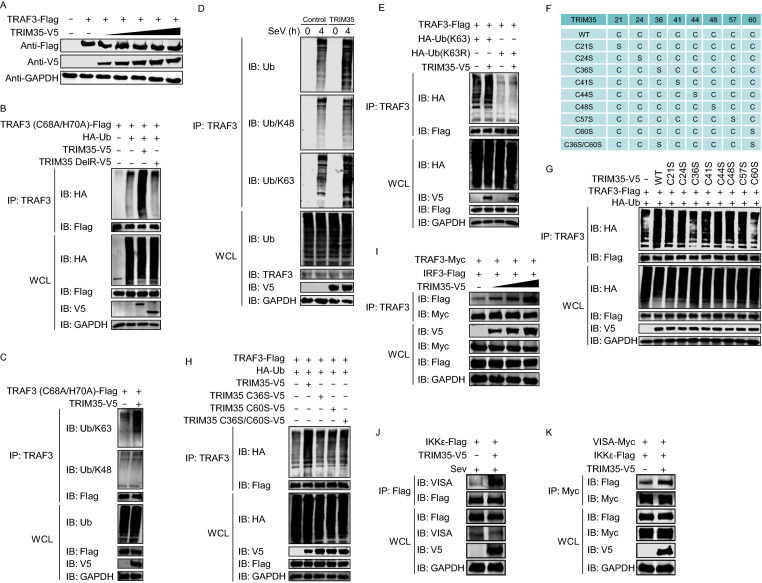
**TRIM35 promotes K63-linked polyubiquitination of TRAF3**. (A) IB analysis of HEK293T cells expressing TRAF3-Flag and increasing amounts of TRIM35-V5. (B) Co-IP and IB analysis to assess TRAF3 ubiquitination in HEK293T cells expressing TRAF3 (C68A/H70A)-Flag, HA-ubiquitin, with or without V5-tagged TRIM35 or its DelRING mutant. (C) Co-IP and IB analysis to assess TRAF3 ubiquitination in HEK293T cells expressing TRAF3 (C68A/H70A)-Flag, with or without TRIM35-V5. (D) Co-IP and IB analysis to assess endogenous TRAF3 ubiquitination in stable TRIM35-overexpressing or control THP-1 cells infected with SeV at the indicated timepoints. (E) Co-IP and IB analysis to assess TRAF3 ubiquitination in HEK293T cells expressing TRAF3-Flag, with or without TRIM35-V5, HA-ubiquitin (K63), and HA-ubiquitin (K63R). (F) Mutants of human TRIM35 in which different cysteine residues were replaced. (G and H) Co-IP and IB analysis to assess TRAF3 ubiquitination in HEK293T cells expressing TRAF3-Flag, HA-ubiquitin, and V5-tagged TRIM35 or its mutants as in (F). (I) Co-IP and IB analysis of HEK293T cells expressing TRAF3-Myc, IRF3-Flag, along with increasing amounts of TRIM35-V5. (J) Co-IP and IB analysis of HEK293T cells expressing IKKε-Flag, with or without TRIM35-V5, followed by infection with SeV for 12 h. (K) Co-IP and IB analysis of HEK293T cells expressing IKKε-Flag, VISA-Myc, with or without TRIM35-V5. Data are representative of at least three independent experiments

K63-linked ubiquitination of TRAF3 is required for induction of type I interferon (Mao et al., 2010; Wang et al., 2013). TRIM35 associates with TRAF3 in the mitochondria and is a RING-type E3 ubiquitin ligase reported to have ubiquitination activity (Chen et al., 2015). We observed that expression of increasing amounts of TRIM35 in HEK293T cells had no effect on the expression level of TRAF3 (Fig. 5A). To determine whether TRIM35 plays a role in the ubiquitination of TRAF3, we used a TRAF3 mutant deficient in E3 ubiquitin ligase activity (C68A/H70A); this mutant is unable to mediate ubiquitination of substrates, including its own auto-ubiquitination (Tseng et al., 2010). TRIM35-mediated polyubiquitination of TRAF3 was readily detected in HEK293T cells expressing exogenous Flag-tagged TRAF3 (C68A/H70A), HA-tagged ubiquitin, and V5-tagged TRIM35 (Fig. 5B). A TRIM35 deletion mutant lacking the RING domain, however, lost the ability to enhance TRAF3 ubiquitination (Fig. 5B).

To determine whether the TRIM35-mediated polyubiquitination of TRAF3 was K48- or K63-linked, we transfected HEK293T cells for the expression of TRAF3 (C68A/H70A)-Flag and TRIM35-V5, and then detected TRAF3 ubiquitination with K48- or K63-linkage-specific polyubiquitin antibodies. We found that TRIM35 promoted K63- but not K48-linked polyubiquitination of TRAF3 (Fig. 5C). To examine the TRIM35-induced ubiquitination of endogenous TRAF3, we measured the ubiquitination of TRAF3 in stable TRIM35-overexpressing THP-1 cells or control cells that were infected with SeV. Endogenous TRAF3 was markedly ubiquitinated with K63-linked ubiquitin chains in TRIM35-overexpression cells compared with the control cells, whereas no difference in the amount of K48-linked ubiquitination of TRAF3 was observed (Fig. 5D). To further confirm the polyubiquitination of TRAF3 by TRIM35 via the K63-linkage, HEK293T cells were co-transfected with TRAF3-Flag plus a ubiquitin mutant in which all lysine residues except K63 were mutated to arginine (K63) or a mutant in which only the K63 residue was mutated to arginine (K63R). Co-IP analyses showed that TRIM35 promoted the ubiquitination of TRAF3 in the presence of the K63-only ubiquitin mutant but not the ubiquitin (K63R) mutant (Fig. 5E). These data clearly demonstrate that TRIM35 induces K63-linked polyubiquitination of TRAF3.

The cysteine (C) residues in the RING domain of TRIM proteins are essential for the E3 ligase activity of these proteins (Li et al., 2014). TRIM35 contains eight cysteine residues in the RING domain. To determine which cysteine residues in the RING domain of TRIM35 are important for the polyubiquitination of TRAF3, we constructed TRIM35 mutants containing serine (S) substitutions in one or two of the eight cysteine residues of the RING domain (Fig. 5F). Co-IP analyses showed that two TRIM35 mutants, C36S and C60S, displayed remarkably reduced polyubiquitination of TRAF3 compared with wild-type TRIM35 (Fig. 5G). Additionally, the single C36S or C60S mutation was sufficient to abolish the E3 ligase activity of TRIM35 in catalyzing the polyubiquitination of TRAF3, since double mutants produced no additive effect (Fig. 5H). These data confirm that the K63-linked polyubiquitination of TRAF3 is catalyzed by TRIM35.

TRIM35-mediated K63-linked polyubiquitination of TRAF3 could regulate the interactions between TRAF3 and other innate immune signaling molecules. To test this hypothesis, we examined the interaction between TRAF3 and IRF3 with or without TRIM35 overexpression. We found that when coexpressed with TRIM35, more IRF3 was co-immunoprecipitated by a mouse anti-TRAF3 mAb, whereas the protein levels of TRAF3 and IRF3 were equal in the cell lysates regardless of the absence or presence of exogenous TRIM35 (Fig. 5I). IKKε is recruited to the C-terminal region of VISA following RNA virus infection, an event that is critical for the transduction of antiviral signaling. We found that the interaction between VISA and IKKε was remarkably increased when co-transfected with TRIM35 (Fig. 5J and 5K). These results indicate that the K63-linked polyubiquitination of TRAF3 catalyzed by TRIM35 is important for the recruitment of the TBK1-IKKε kinase complex and the activation of RIG-I signaling.

### IAV PB2 impedes K63-linked polyubiquitination of TRAF3 and disrupts the formation of the VISA–TRAF3 complex

**Figure 6:**
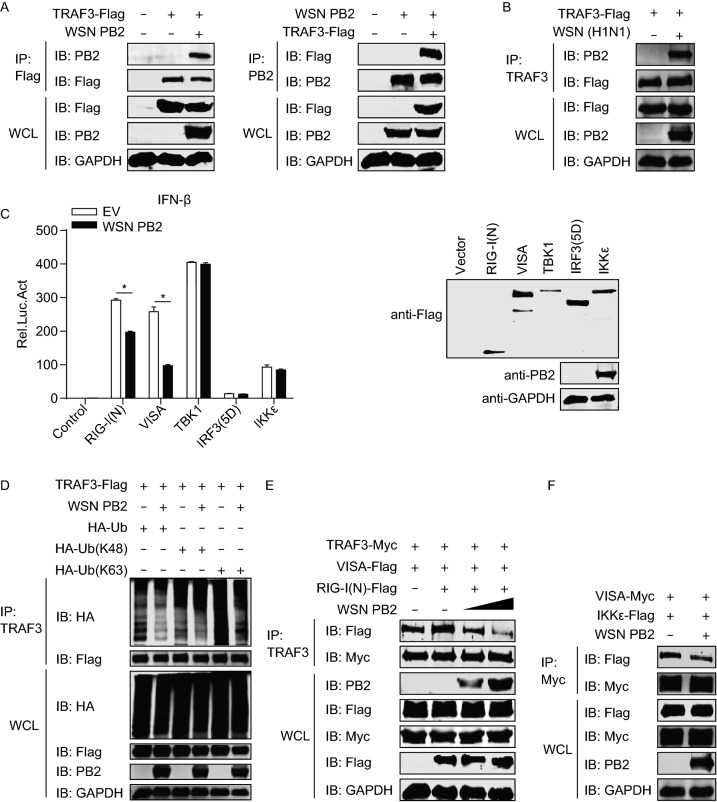
**IAV PB2 interferes with K63-linked polyubiquitination of TRAF3 and the formation of the VISA-TRAF3 complex**. (A) Co-IP and IB analysis of HEK293T cells expressing TRAF3-Flag and WSN (H1N1) PB2. (B) Co-IP and IB analysis of HEK293T cells expressing TRAF3-Flag for 36 h, followed by infection with WSN (H1N1) virus for 12 h. (C) IFN-β promoter luciferase reporter assay of HEK293T cells transfected for 48 h with plasmid expressing WSN (H1N1) PB2 or empty vector, along with a construct expressing Flag-tagged RIG-I(N), VISA, TBK1, IKKε, or IRF3(5D). Results are expressed relative to *Renilla* luciferase activity (left panel). IB analysis was performed to assess the expression of the transfected constructs (right panel). (D) Co-IP and IB analysis to assess TRAF3 ubiquitination in HEK293T cells expressing TRAF3-Flag, with or without WSN (H1N1) PB2, along with HA-ubiquitin, HA-ubiquitin (K48) or HA-ubiquitin (K63). (E) Co-IP and IB analysis of HEK293T cells expressing TRAF3-Myc, VISA-Flag, RIG-I(N)-Flag, along with increasing amounts of WSN (H1N1) PB2. (F) Co-IP and IB analysis of HEK293T cells expressing VISA-Myc, IKKε-Flag, with or without WSN (H1N1) PB2. Data are representative of at least three independent experiments. Means ± SD are shown in (C, left panel) (*n* = 3). Two-tailed unpaired t-test was used for the statistical analysis, *P < 0.05

TRAF3 is a key molecule in the activation of RIG-I-mediated antiviral innate immunity (Saha et al., 2006). We hypothesized that IAV infection could target TRAF3 to antagonize RIG-I signaling. To test this concept, we first performed co-IP experiments to examine the potential interaction between IAV proteins and TRAF3. We found that among the ten essential IAV proteins, only PB2 interacted with TRAF3 (Figs. 6A and S3A–J). The co-IP assay performed with HEK293T cells that were transfected with plasmids to express TRAF3-Flag and then infected with WSN (H1N1) virus, revealed the interaction between PB2 and TRAF3 during the viral infection course (Fig. 6B). Further co-IP assays with a series of PB2 truncation mutants indicated that the C-terminal 490–759 region of PB2 was critical for PB2 binding with TRAF3 (Fig. S3K). Similarly, we found that the C-terminal MATH domain of TRAF3 was responsible for mediating the interaction with IAV PB2 (Fig. S3L and S3M).

We next investigated the effect of IAV PB2 on various components of the RIG-I signaling pathway. HEK293T cells were co-transfected with plasmids expressing IAV PB2 and different RIG-I signaling components, including RIG-I(N), VISA, TBK1, IKKε and the active form of IRF3 [IRF3(5D)], together with a luciferase reporter construct driven by the IFN-β promoter. The luciferase reporter assay showed that IAV PB2 inhibited the IFN-β promoter activity induced by RIG-I(N) or VISA, but not by TBK1, IKKε, or IRF3(5D) (Fig. 6C), indicating that TRAF3 is the target of IAV PB2. These results, together with the above data, demonstrate that IAV PB2 interacts with TRAF3, and counteracts RIG-I antiviral signaling by targeting TRAF3.

To determine how IAV PB2 inhibits RIG-I antiviral signaling through its interaction with TRAF3, we transfected HEK293T cells with plasmids for the expression of TRAF3 in combination with IAV PB2 and ubiquitin. Co-IP experiments indicated that co-expression of IAV PB2 decreased the ubiquitination level of TRAF3 (Fig. 6D). We then investigated the type of TRAF3 polyubiquitination inhibited by IAV PB2. By replacing ubiquitin with its K48 (all lysine residues except K48 were mutated to arginine) or K63 mutant in the co-IP experiment, we found that IAV PB2 inhibited K63-linked polyubiquitination of TRAF3 but had no role in the K48-linked polyubiquitination of TRAF3 (Fig. 6D).

TRAF3 binds to a functional site in the proline-rich region of VISA (Saha et al., 2006), which is critical for IFN production upon viral infection. To examine whether IAV PB2 affects IFN signaling at the level of the VISA-TRAF3 interaction, we examined the association of VISA with TRAF3 in the presence of IAV PB2. We found that VISA interacted with TRAF3 and that the level of this interaction was increased by the co-expression of RIG-I(N). However, IAV PB2 markedly disrupted this interaction in a dose-dependent manner (Fig. 6E). Moreover, the interaction between VISA and IKKε was remarkably decreased upon co-transfection with IAV PB2 (Fig. 6F).

Collectively, these results demonstrate that IAV PB2 inhibits the K63-linked polyubiquitination of TRAF3 and disrupts the formation of the VISA-TRAF3 complex, which, in turn, suppresses IFN-β production.

### TRIM35 targets IAV PB2 for K48-linked polyubiquitination and degradation

**Figure 7:**
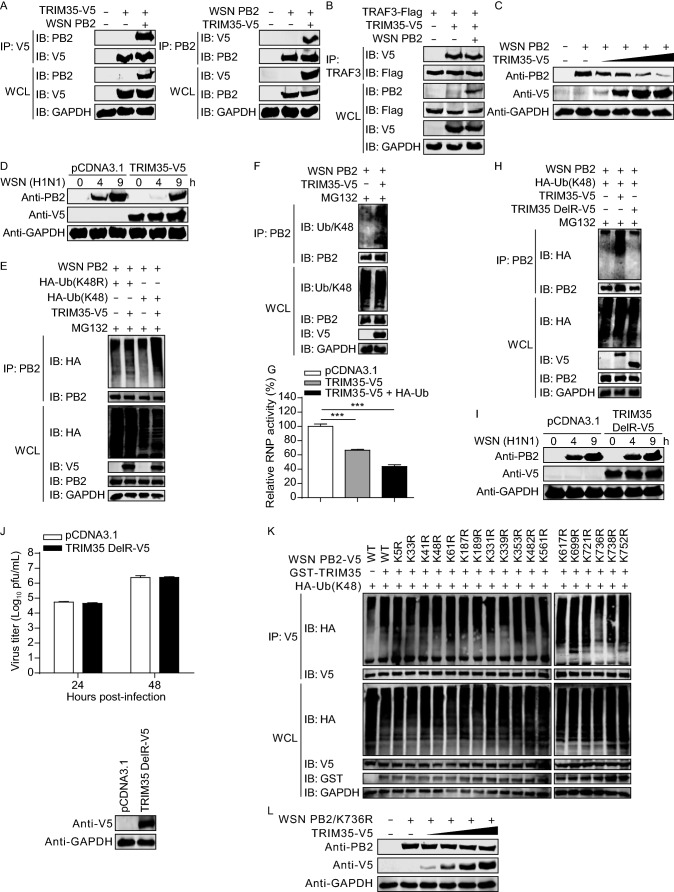
**TRIM35 catalyzes K48-linked polyubiquitination and degradation of IAV PB2**. (A) Co-IP and IB analysis of HEK293T cells expressing TRIM35-V5 and WSN (H1N1) PB2. 10 μg/mL MG132 was added into cells at 6 h post-transfection (right panel). (B) Co-IP and IB analysis of HEK293T cells expressing TRAF3-Flag, TRIM35-V5, with or without WSN (H1N1) PB2. (C) IB analysis of HEK293T cells expressing WSN (H1N1) PB2 and increasing amounts of TRIM35-V5. (D) IB analysis of HEK293T cells expressing TRIM35-V5 or control vector for 36 h, followed by infection with WSN (H1N1) virus. (E) Co-IP and IB analysis to assess WSN (H1N1) PB2 ubiquitination in HEK293T cells expressing WSN (H1N1) PB2, with or without TRIM35-V5, along with HA-ubiquitin (K48) or HA-ubiquitin (K48R). (F) Co-IP and IB analysis to assess WSN (H1N1) PB2 ubiquitination in HEK293T cells expressing WSN (H1N1) PB2, with or without TRIM35-V5. (G) Minigenome assay in HEK293T cells expressing WSN (H1N1) PB2, PB1, PA, and NP, pHH21-SC09NS F-Luc, pRL-TK, along with control vector, TRIM35-V5, or both TRIM35-V5 and HA-ubiquitin. Results are expressed relative to *Renilla* luciferase activity. (H) Co-IP and IB analysis to assess WSN (H1N1) PB2 ubiquitination in HEK293T cells expressing WSN (H1N1) PB2, HA-ubiquitin (K48), along with V5-tagged TRIM35 or TRIM35 DelRING mutant. (I) IB analysis of HEK293T cells expressing V5-tagged TRIM35 DelRING mutant or control vector for 36 h, followed by infection with WSN (H1N1) virus. (J) Replication of WSN (H1N1) virus (MOI = 0.1) in HEK293 cells transfected for 24 h to express V5-tagged TRIM35 DelRING mutant or control vector, determined by plaque assay on MDCK cells. IB analysis was shown in the bottom panel. (K) Co-IP and IB analysis to assess K48-linked ubiquitination of WSN (H1N1) PB2 or its mutants in HEK293T cells expressing V5-tagged WSN (H1N1) PB2 or its mutants, GST-TRIM35, and HA-ubiquitin (K48). (L) IB analysis of HEK293T cells expressing WSN (H1N1) PB2 K736R mutant and increasing amounts of TRIM35-V5. Data are representative of at least three independent experiments. Means ± SD are shown in (G) and (J, upper panel) (*n* = 3). Two-tailed unpaired *t*-test was used for the statistical analysis, ***P < 0.001

In addition to regulating antiviral innate immunity, TRIM proteins are involved in the counteraction of viral infection by targeting viral proteins (Rajsbaum et al., 2014). We therefore investigated whether TRIM35 interacts with any IAV proteins. Transient transfection and co-IP experiments indicated that TRIM35 interacted with PB2, but not with any other essential proteins of WSN (H1N1) virus (Figs. 7A and S4A–J). Consistent with this finding, the interaction was also observed between TRIM35 and the PB2 protein of two other IAV strains, A/Anhui/2/2005 (AH05, H5N1) and A/Anhui/1/2013 (AH13, H7N9) (Fig. S4K), demonstrating the universality of this interaction. The TRIM35-PB2 interaction was also observed in WSN (H1N1)-infected cells (Fig. S4L).

Domain-mapping experiments indicated that deletion of the TRIM35 PRY/SPRY domain abolished the interaction of TRIM35 with IAV PB2 (Fig. S4M). Furthermore, the single PRY/SPRY domain of TRIM35 was sufficient to mediate the interaction of TRIM35 with IAV PB2 (Fig. S4N). Together, these results indicate that TRIM35 interacts with PB2 through the C-terminal PRY/SPRY domain.

To investigate the biological effect of the TRIM35/IAV PB2 interaction, we first determined whether TRIM35 and PB2 compete with each other for their common binding partner, TRAF3. Co-IP experiments indicated that the co-expression of IAV PB2 did not affect the interaction between TRIM35 and TRAF3 (Fig. 7B), indicating that the interactions among these three proteins are non-competitive.

TRIM proteins are often involved in the degradation of target proteins through their ubiquitin E3 ligase activity (Hatakeyama, 2017). We therefore explored whether TRIM35 could degrade IAV PB2. Co-expression of gradually increasing amounts of TRIM35 decreased the level of IAV PB2 in a dose-dependent manner (Fig. 7C). We also found that pre-overexpression of TRIM35 in HEK293T cells led to reduced levels of PB2 during WSN (H1N1) infection (Fig. 7D).

Because proteins modified with K48-linked polyubiquitin are classic targets for proteasomal degradation (Rieser et al., 2013), we examined whether TRIM35 could catalyze the K48-linked polyubiquitination of IAV PB2. HEK293T cells were transfected for the expression of WSN (H1N1) PB2, TRIM35, and a K48 ubiquitin mutant or a ubiquitin mutant in which only the K48 residue was mutated to arginine (K48R). As shown in Fig. 7E, TRIM35 conjugated K48 ubiquitin onto PB2, whereas K48R ubiquitin was not coupled onto PB2, indicating that TRIM35 catalyzed the K48-linked polyubiquitination of IAV PB2. Similarly, we found that TRIM35 promoted the polyubiquitination of PB2 with K48-linkage by the use of endogenous ubiquitin (Fig. 7F). TRIM35-induced K48-linked polyubiquitination also led to the degradation of the PB2 protein of AH05 (H5N1) and AH13 (H7N9) virus (Fig. S5A and S5B).

PB2, PB1, PA, and nucleoprotein (NP), together with viral RNA, constitute the viral ribonucleoprotein (vRNP) complex, which is the functional unit for the transcription and replication of the viral genome (Arranz et al., 2012; Moeller et al., 2012). To determine whether TRIM35-mediated PB2 degradation has an effect on the vRNP complex, we performed a minigenome assay in which HEK293T cells were co-transfected with constructs for the expression of TRIM35 and the vRNP complex proteins (PB2, PB1, PA, and NP), along with a vRNA-like luciferase reporter. As shown in Fig. 7G, the co-expression of TRIM35 led to a decrease in vRNP activity, and the inclusion of exogenous ubiquitin further reduced the vRNP activity. These results indicate that TRIM35-mediated K48-linked polyubiquitination and degradation of IAV PB2 protein inhibit the transcription and replication of the viral genome.

To determine whether the E3 ligase activity of TRIM35 is essential for the K48-linked polyubiquitination of IAV PB2, WSN (H1N1) PB2, K48 ubiquitin mutant, and TRIM35 or its DelRING mutant (the RING domain was deleted) were expressed in HEK293T cells. As shown in Fig. 7H, TRIM35 increased the levels of K48-linked polyubiquitination of PB2, whereas ectopic expression of the DelRING TRIM35 mutant had no effect on PB2 ubiquitination. We also found that compared with wild-type TRIM35 (Fig. 7D), the DelRING TRIM35 mutant was unable to reduce PB2 expression levels during IAV infection (Fig. 7I). We next infected DelRING TRIM35-overexpressing HEK293 cells or control cells with WSN (H1N1) virus, and found that the production of infectious virus in the culture supernatants was comparable between the two cell types at 24 and 48 h p.i. (Fig. 7J), indicating that the inhibitory role of TRIM35 in IAV growth was abolished by the deletion of the RING domain.

Ubiquitination involves the attachment of ubiquitin to acceptor lysine residues on substrate proteins (Sadowski and Sarcevic, 2010). Eighteen lysine residues have been reported to be conserved among IAV PB2 proteins (Zhang et al., 2017). To identify the specific PB2 lysine residues ubiquitinated by TRIM35, we generated PB2 mutants containing individual lysine to arginine mutations. We found that the ubiquitination level of one PB2 mutant, K736R, was dramatically reduced compared with that of wild-type PB2 and the other PB2 mutants (Fig. 7K), indicating that residue K736 is the major ubiquitination site on IAV PB2. As expected, co-expression of increasing amounts of TRIM35 lost the effect on the expression level of the PB2 K736R mutant (Fig. 7L), compared with that of the wild-type PB2 (Fig. 7C). Notably, K736 is highly conserved in the PB2 of IAVs (Table S1), suggesting that TRIM35-mediated polyubiquitination and degradation of PB2 is a common host defense mechanism against IAV infection.

Taken together, our data indicate that TRIM35 promotes K63-linked polyubiquitination of TRAF3, leading to enhanced type I IFN production and inhibition of viral replication; TRIM35 also mediates K48-linked polyubiquitination and degradation of IAV PB2, and counteracts PB2-mediated suppression of K63-linked polyubiquitination of TRAF3. Synergies between the two mechanisms lead to a dampened replication and virulence of IAV.

## Discussion

The innate immune system uses pattern recognition receptors (PRRs) in different cellular compartments to recognize the conserved pathogen-associated molecular patterns (PAMPs) of invading pathogens (Kawai and Akira, 2006; Thompson et al., 2011). Many of these PRRs have been well characterized, including RIG-I. RIG-I senses cytoplasmic viral RNA and activates innate immune signaling cascades and the secretion of type I IFN (Kawai and Akira, 2006; Thompson et al., 2011). TRAF3 is a key adaptor in the RIG-I signaling pathway (Saha et al., 2006). During viral infection, K63-linked polyubiquitination of TRAF3 leads to the recruitment of TBK1-IKKε and subsequent activation of IRF3 (Fitzgerald et al., 2003; Hacker et al., 2011). Here we provide several lines of evidence to demonstrate that TRIM35 serves as an important regulator of RIG-I-mediated antiviral innate immunity through direct conjugation of K63-linked ubiquitin chains to TRAF3. Consistently, cells expressing TRIM35 (C36S, or C60S) lost the ability to catalyze K63-linked ubiquitination of TRAF3. Thus, our study has revealed novel insights into the role of TRIM35 in the activation of RIG-I-mediated innate immunity.

TRIM35 is ubiquitously expressed in different tissues and was initially implicated in the apoptotic process (Kimura et al., 2003). Regarding its role in innate immunity, TRIM35 has been reported to negatively regulate toll-like receptor 7/9 (TLR7/9)-mediated type I IFN production by inducing interferon regulatory factor 7 (IRF7) degradation (Wang et al., 2015). In the present study, however, we found that TRIM35 is a positive regulator of RIG-I-mediated innate immunity, leading to enhanced production of type I IFN by facilitating K63-linked polyubiquitination of TRAF3. The difference between our results and those of the cited study suggests that TRIM35 plays different roles in different innate immune signaling pathways. In our study, the expression of TRIM35 was shown to be critical for type I IFN response triggered by poly(I:C), 5′-pppRNA or SeV, which are the prototypic ligands that potently stimulate the RIG-I-mediated induction of the type I IFN pathway. Consistent with in vitro findings, the depletion of *Trim35* led to an impaired yield of IFN-β in the serum of VSV-infected *Trim35*^−/−^ mice, and *Trim35*^−/−^ mice were more susceptible to IAV infection than were their wild-type counterparts.

We found that the expression of TRIM35 was dramatically induced upon infection with IAV or SeV, or stimulation with poly(I:C), 5′-pppRNA or IFN- IFN-β. This suggests that TRIM35 may form a positive feedback loop with type I IFN in which the increased yield of type I IFN facilitates the expression of downstream effectors, e.g., TRIM35, whose expression in turn further promotes the production of type I IFN.

Given the central role of IFN in the host innate immune response to viral infection, IAV has adopted several different strategies to escape from the IFN responses. Both viral PB2 and polymerase basic protein 1-frame 2 (PB1-F2) associate with VISA to suppress the production of type I IFN (Graef et al., 2010; Varga et al., 2011). Other viral proteins, such as PB1, PA, and nonstructural protein 1 (NS1), also act to impair RIG-I-mediated antiviral immunity (Li et al., 2006; Mibayashi et al., 2007; Jiao et al., 2008; Zhu et al., 2008; Gack et al., 2009; Iwai et al., 2010; Liedmann et al., 2014; Yi et al., 2017). In the present study, we found that IAV PB2 suppressed the K63-linked ubiquitination of TRAF3, and impaired the formation of the VISA-TRAF3 complex, thereby hindering the downstream transduction of the RIG-I signaling cascade. This finding is in agreement with previous reports that the formation of TRAF3-VISA signaling complex could possibly be affected by the ubiquitination status of TRAF3 (Zhu et al., 2015; Qian et al., 2017). Our results, together with those of others (Graef et al., 2010), reveal that IAV PB2 utilizes distinct means to target different elements of the RIG-I signaling pathway. This tactic could expand the toolbox of the viral PB2 protein to counteract the host defense system and confer advantages for replication and virulence.

Different types of ubiquitin chains covalently attached to target proteins have emerged as important ways to regulate protein functions. K48-linked polyubiquitination is a signal for proteasomal degradation of target proteins, whereas K63-linked polyubiquitination is a non-proteolytic mode of modification that is important for the assembly and function of protein signaling complexes (Pickart and Fushman, 2004; Rieser et al., 2013). Like most TRIM proteins, TRIM35 contains an N-terminal RING domain and can function as an E3 ubiquitin ligase. Our study revealed that the E3 ligase activity of TRIM35 is essential for its antiviral activity. Intriguingly, TRIM35 employs two means of ubiquitination in the antiviral immune response to IAV infection: the K63-linked polyubiquitination of TRAF3 promotes the activation of the RIG-I antiviral signaling cascade and the production of type I IFN; the K48-linked polyubiquitination of viral PB2 leads to the direct proteasomal degradation of PB2, an essential component of the vRNP complex that is responsible for the transcription and replication of IAV genome, and also counteracts PB2-mediated suppression of K63-linked polyubiquitination of TRAF3. Moreover, we identified K736 in PB2 as the target lysine residue for TRIM35-mediated K48-linked polyubiquitination and degradation. The utilization of these two types of ubiquitination linkages by TRIM35 could maximize its ability to antagonize IAV infection.

**Figure 8:**
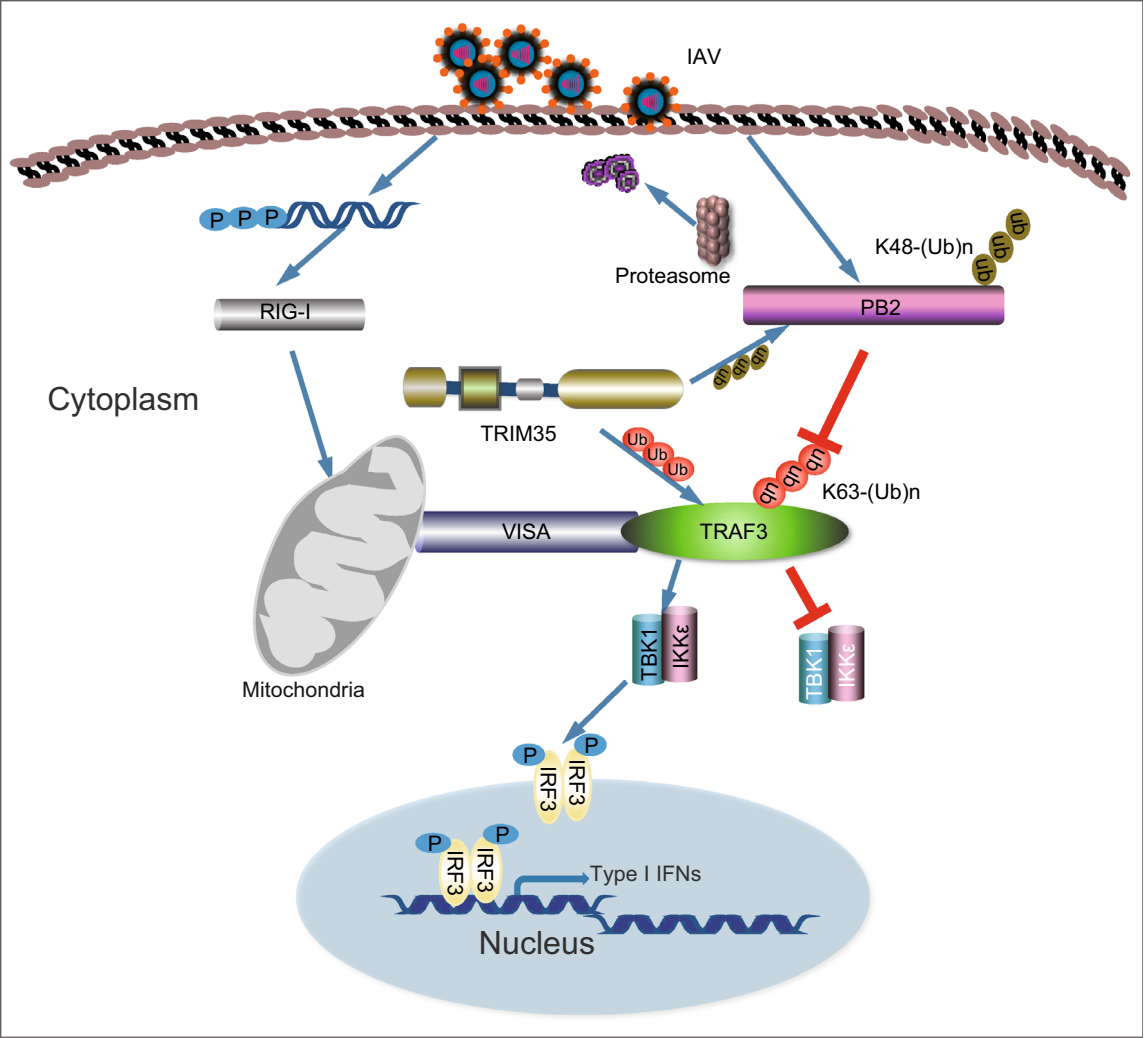
**Schematic depiction of the proposed role of TRIM35 in antiviral immunity against IAV infection**. TRIM35, TRAF3, and IAV PB2 form a circuit in which two means are employed by TRIM35 to defend the host against IAV infection. TRIM35 catalyzes K63-linked polyubiquitination of TRAF3 and activates RIG-I antiviral signaling; although IAV PB2 prevents K63-linked polyubiquitination of TRAF3, TRIM35 directly mediates K48-linked polyubiquitination and degradation of IAV PB2

In summary, here we demonstrate that a circuit is formed among TRIM35, TRAF3, and IAV PB2 (Fig. 8). In this circuit, PB2 prevents K63-linked ubiquitination of TRAF3, whereas TRIM35 defends the host against IAV infection by catalyzing K63-linked polyubiquitination of TRAF3 and activating RIG-I antiviral signaling, and by the direct K48-linked polyubiquitination and degradation of viral PB2. Exploitation of this natural defense mechanism may offer more effective strategies for controlling IAV infections.

## Methods

### Mice

*Trim35*^−/−^ mice were generated by Shanghai Model Organisms Center, Inc (Shanghai, China). Genotyping of wild-type and knockout mice was performed with the following primers: forward primer 5′-CCAGTATGACTCTTACCTCTTCCG-3′ and reverse primer 5′-CAGGGTTTCTTCTGTGTCTCCA-3′ for wild-type mice; forward primer 5′-GTCTACAGTCATGCTACCTT-3′ and reverse primer 5′-GTCTTCTCACTCACTGCATT-3′ for *Trim35*^−/−^ mice. All mice were on the C57BL/6J background and were maintained under specific-pathogen-free conditions. All mice used were female and 6 weeks of age. All animal experiments were reviewed and approved by the Review Board of the Harbin Veterinary Research Institute (HVRI) of the Chinese Academy of Agricultural Sciences (CAAS).

### Antibodies and reagents

5′-pppRNA and poly(I:C) were purchased from InvivoGen, and were used at a final concentration of 0.5 µg/mL and 10 µg/mL, respectively. MG132, IFN-β, IFN-β ELISA kit, Glutathione-Sepharose, and the Dual-Luciferase Reporter Assay System were purchased from Sigma Aldrich, R&D Systems, PBL Interferon Source, GE Healthcare, and Promega, respectively. Antibodies against the following IAV proteins were generated in our Laboratory: PB2, PB1, PA, and NP. Anti-TRAF3 (sc-6933), anti-TRIM35 (sc-100880), and anti-ubiquitin (sc-8017) were from Santa Cruz. Anti-TRAF3 (4729), anti-MAVS (24930), anti-K63-linkage specific polyubiquitin (5621), anti-K48-linkage specific polyubiquitin (8081) were from Cell Signaling. Anti-Flag (F1804; F7425), anti-Myc (C3965; M4439), anti-V5 (V8012), and anti-TRIM35 (SAB2103161) were from Sigma Aldrich. Anti-IFITM3 (11714-1-AP), anti-HA (51064-2-AP), and anti-GAPDH (10494-1-AP) were from Proteintech. Anti-V5 (AB3792) was from Millipore. Anti-GFP (ab6556), and anti-GST (ab58626) were from Abcam. Mouse IgG (A7028) was from Beyotime. Alexa Fluor 633 goat anti-mouse IgG (H + L) (A21050) and Alexa Fluor 488 donkey anti-rabbit IgG (H + L) (A21206) from Life Technologies were used for confocal microscopy. DyLight 800 goat anti-mouse IgG (H + L) (072-07-18-06) and DyLight 800 goat anti-rabbit IgG (H + L) (072-07-15-06) were from KPL.

### Cells and viruses

HEK293, HEK293T, A549, THP-1, and MDCK cells were cultured as previously described (Zhu et al., 2017). Hela cells were cultured in DMEM (Life Technologies) containing 10% fetal bovine serum (Sigma-Aldrich). RAW264.7 cells were cultured in RPMI 1640 medium (Life Technologies) with 10% fetal bovine serum. Peritoneal macrophages were harvested from mice 4 days after thioglycollate (BD Biosciences) injection. A/WSN/33 (WSN, H1N1), vesicular stomatitis virus (VSV), and VSV-GFP virus were stored in our laboratory. Sendai virus (SeV) was provided by J. Tian (HVRI, China).

### Plasmids and transfections

The ISRE promoter luciferase reporter plasmid was provided by C. Weng (HVRI, China). The IFN-β promoter luciferase reporter plasmid was purchased from Addgene. *TRIM* cDNAs (shown in Fig. 1A) were purchased from Thermo Scientific, amplified by standard PCR, and cloned into pCDNA3.1 with a V5 tag at the C-terminus. *TRIM35* cDNA was also cloned into pCAGGS (provided by Y. Kawaoka, University of Wisconsin-Madison) with a GST tag at the N-terminus. Open reading frames (ORFs) of *PB2* from WSN (H1N1), AH05 (H5N1), or AH13 (H7N9) virus, as well as *PB1*, *PA*, and *NP* of WSN (H1N1) virus, were cloned into pCAGGS. ORFs of *RIG-I*, *VISA*, *TRAF3*, *TBK1*, *IKKε*, and *IRF3,* which were amplified from HEK293T or A549 cells, and *HA*, *NP*, *NA*, *M1*, and *NS1* of WSN (H1N1) virus were cloned into pCAGGS with a Flag tag at the C-terminus. ORFs of the *M2* and *NS2* genes of WSN (H1N1) virus were cloned into the pEGFP-C1 vector (Clontech). ORFs of *VISA* and *TRAF3* were also inserted into pCAGGS with a Myc tag at the C-terminus. His-tagged TRAF3 was cloned into the pET30a vector. Deletion, truncation, or point mutations of *TRIM35*, *TRAF3*, and WSN (H1N1) *PB2* were generated by using the Fast Mutagenesis System (Transgen). pHH21-SC09NS F-Luc, used to produce influenza vRNA-like luciferase reporter, has been previously described (Luo et al., 2018; Liang et al., 2019). All plasmid constructs were confirmed by DNA sequencing. SiRNA sequences for transient silencing of human *TRIM35*, mouse *Trim35*, or scrambled siRNA are listed in Table S2. All of these siRNAs were obtained from GenePharma. For transient transfection of plasmids or siRNA duplexes into A549, HEK293, HEK293T, or RAW264.7 cells, Lipofectamine 2000 reagent (Invitrogen) was used.

### Immunoblot analysis and co-immunoprecipitation

For immunoblot analysis, cells or tissues were lysed with Pierce IP lysis buffer [1% (*v*/*v*) NP-40, 50 mmol/L Tris-HCl, 50 mmol/L EDTA, 150 mmol/L NaCl, pH 7.4] supplemented with a protease inhibitor cocktail (Roche). Protein concentrations in the extracts were measured with a bicinchoninic acid assay kit (Pierce) and were made equal across samples by adding extraction reagent. For immunoprecipitation, whole cell lysates were collected 48 h after transfection or at the indicated timepoints post-infection, and lysed in IP buffer. After centrifugation for 10 min at 14,000 ×*g*, supernatants were incubated with protein G-Agarose immunoprecipitation reagent (Roche) together with the corresponding primary antibodies. After 6 h of incubation, beads were washed four times with IP buffer. Immunoprecipitates or whole cell lysates were boiled with SDS sample buffer, separated by sodium dodecyl sulfate-polyacrylamide gel electrophoresis (SDS-PAGE), transferred onto nitrocellulose membranes, and then blotted with specific antibodies.

### GST pull-down

His-TRAF3 was expressed in BL21 (DE3) cells and purified by using His-Trap FF (GE Healthcare). HEK293T cells grown in 10-cm dishes were transfected with 10 μg of pCAGGS-GST-TRIM35. At 48 h post-transfection, cells were lysed with 300 μL of IP buffer. The lysates were mixed with 40 μL of Glutathione Sepharose 4 Fast Flow (GE Healthcare) and rocked for 4 h at 4 °C. After three washes, purified His-TRAF3 was added and incubated for 2 h at 4 °C. After three more washes, the bound proteins were separated by SDS-PAGE and detected by immunoblot analysis.

### Generation of a stable TRIM35-overexpressing THP-1 cell line

The human *TRIM35* gene was cloned into the pLVX-ISRE-ZsGreen1 vector (Clontech). HEK293T cells cultured in 10-cm dishes were transfected with either pLVX-TRIM35 or with the empty pLVX vector by using Lipofectamine 2000 reagent. At 48 h post-transfection, the lentiviruses produced were collected and used to infect THP-1 cells in the presence of 8 mg/mL polybrene. At 3 days p.i., the surviving cells were sorted on a MoFlo XDP cell sorter (Beckman Coulter) with ZsGreen as a marker. The sorted cells were individually cloned into 96-well plates, propagated, and examined for TRIM35 overexpression by quantitative reverse-transcription PCR (qRT-PCR) and immunoblot analysis.

### Ubiquitination assay

To analyze the effect of TRIM35 on the ubiquitination of TRAF3 in transfected cells, HEK293T cells were transfected with the indicated plasmids expressing TRAF3 (C68A/H70A)-Flag or TRAF3-Flag, TRIM35-V5 or its mutants, in the presence or absence of HA-tagged ubiquitin or its mutants, and then whole cell lysates were immunoprecipitated with the anti-TRAF3 antibody and analyzed by immunoblot analysis with anti-HA, anti-Flag, anti-ubiquitin (K48), or anti-ubiquitin (K63) antibodies. To analyze the effect of TRIM35 on the ubiquitination of endogenous TRAF3, stable TRIM35-overexpressing THP-1 cells or control cells were infected with SeV, then whole cell lysates were immunoprecipitated with anti-TRAF3 antibody, and analyzed by immunoblot analysis with anti-ubiquitin, anti-ubiquitin (K48), or anti-ubiquitin (K63) antibodies.

To analyze the effect of WSN (H1N1) PB2 on the ubiquitination of TRAF3, HEK293T cells were transfected with plasmids expressing TRAF3-Flag and WSN (H1N1) PB2, in the presence or absence of HA-tagged ubiquitin or its mutants, and then whole cell lysates were immunoprecipitated with the anti-TRAF3 antibody and analyzed by immunoblot analysis with anti-HA or anti-Flag antibodies.

To analyze the effect of TRIM35 on the ubiquitination of WSN (H1N1) PB2 in transfected cells, HEK293T cells were transfected with plasmids expressing WSN (H1N1) PB2, TRIM35-V5 (or GST-TRIM35) or its mutants, and HA-tagged ubiquitin (K48) or ubiquitin (K48R). Where indicated, 10 μg/mL MG132 was added into cells at 6 h post-transfection. Whole cell lysates were then immunoprecipitated with the anti-PB2 or anti-V5 antibody and analyzed by immunoblot analysis with anti-PB2, anti-HA, anti-V5, or anti-ubiquitin (K48) antibodies.

### Immunofluorescence assay and confocal microscopy

HEK293T or HeLa cells grown on glass-bottom dishes were transfected, treated, and/or infected as indicated. Cells were fixed with 4% (*w*/*v*) paraformaldehyde (PFA) in PBS for 20 min, and permeabilized with 0.1% (*v*/*v*) Triton-X-100 in PBS for 30 min. The permeabilized cells were blocked with 5% bovine serum albumin in PBS for 1 h, and then stained with the indicated primary antibodies, followed by incubation with secondary antibodies conjugated to Alexa Fluor 488 or Alexa Fluor 633. Nuclei were counterstained with DAPI (Sigma-Aldrich). For mitochondrial staining, living cells were incubated with 300 nmol/L Mito Tracker Red (Invitrogen) for 30 min at 37 °C. Imaging of the cells was carried out using a ZEISS laser-scanning confocal microscope.

### Luciferase assays

HEK293T cells were transfected with pcDNA3.1-TRIM35-V5 or empty pcDNA3.1 vector, ISRE-Luc or IFN-β-Luc reporter, and an internal control pRL-TK for 24 h. They were then stimulated with SeV for 12 h, and subsequently lysed for evaluation in the luciferase assay. To analyze the effect of WSN (H1N1) PB2 on the stimulation of RIG-I signaling, HEK293T cells were transfected for the expression of WSN (H1N1) PB2, IFN-β-Luc reporter, an internal control pRL-TK, along with Flag-tagged RIG-I(N), VISA, TBK1, IKKε, or IRF3(5D). At 48 h post-transfection, the cell lysates were prepared for the luciferase assay. To determine the effect of TRIM35 on influenza vRNP activity, HEK293T cells were transfected with empty pcDNA3.1 vector or pcDNA3.1-TRIM35-V5, the four constructs for the expression of the vRNP complex proteins from WSN (H1N1) virus (PB2, PB1, PA, and NP), pHH21-SC09NS F-Luc, and pRL-TK, together with or without the plasmid for the expression of ubiquitin. At 48 h post-transfection, cell lysates were prepared for the luciferase assay.

The luciferase assay was performed by using the dual luciferase reporter assay system on a GloMax 96 microplate luminometer (Promega). Data were normalized for transfection efficiency by calculating the ratio between the firefly luciferase activity and the Renilla luciferase activity.

### Cell viability

Cell viability was assessed by using a CellTiter-Glo luminescent cell viability assay (Promega) as described previously (Zhu et al., 2017). Briefly, A549 cells were transfected with siRNA targeting *TRIM35* or with scrambled siRNA (30 nmol/L). Forty-eight hours later, 100 μL of CellTiter-Glo reagent was added directly into each well for 10 min to induce cell lysis. The luminescence of the cell lysates was measured with a GloMax 96 Microplate Luminometer.

### Enzyme-linked immunosorbent assay (ELISA)

6-week-old female *Trim35*^+/+^ and *Trim35*^*−/−*^ mice (five per group) were infected with 2 × 10^7^ plaque-forming units (PFU) of VSV or were mock infected with PBS by intravenous injection. Serum IFN-β concentrations were measured on day 5 by using ELISA Kits (PBL Interferon Source).

### RNA quantification

Total RNA was extracted with TRIzol reagent (Invitrogen) and was used to synthesize first-strand cDNA with a cDNA synthesis kit (Takara). Specific primers used for real-time PCR are shown in Table S3. Real-time PCR was performed by using the SYBR Green PCR Master Mix (Takara) with a LightCycler 480 II system (Roche). Relative RNA quantities were determined by using the ∆∆Ct method, and were normalized to the expression of the cellular actin gene.

### Viral infection *in vitro* and plaque assays

For experiments involving SeV virus infection, HEK293T, HeLa, THP-1, RAW264.7, or primary mouse peritoneal macrophage cells were infected with SeV (50 HAU/mL) for the indicated periods of times. In an experiment specifically designed to evaluate the effect of TRIM35 on the production of IFN-β, HEK293T cells were transfected with TRIM35-expressing or control plasmids for 24 h, and then infected with SeV for 12 h. The cell supernatants were collected and inactivated with ultraviolet (UV) light. Fresh HEK293T cells were then incubated with the UV-inactivated cell supernatants for 24 h, followed by infection with VSV-GFP at an MOI of 0.1 for 12 h. VSV-GFP replication was analyzed by fluorescence microscopy and immunoblot analysis.

A549, HEK293, and mouse peritoneal macrophage cells were infected with WSN (H1N1) virus at an MOI of 0.1. Supernatants were collected at 24 and 48 h p.i., and virus titers were determined by means of plaque assays on MDCK cells as described previously (Zhu et al., 2017).

### Viral infection *in vivo*

6-week-old female *Trim35*^+/+^ and *Trim35*^−/−^ mice were anesthetized with CO_2_ and intranasally infected with WSN (H1N1) influenza virus (2 × 10^3^ PFU). The body weight and survival of groups of six mice were monitored daily for 14 days. To assess virus replication, the infected mice (six mice per group) were euthanized on day 3 p.i., and their lungs were collected, homogenized, and titrated for infectious virus by use of plaque assays on MDCK cells.

### Lung histopathology

Lungs from WSN (H1N1) virus- or mock-infected mice were collected on day 3 p.i., fixed in 10% phosphate-buffered formalin, embedded into paraffin, sectioned (4-μm thick), stained with hematoxylin and eosin solution, and examined by light microscopy for histopathological changes. For the detection of viral NP antigen, immunohistochemical (IHC) staining was performed by using a rabbit anti-NP polyclonal antibody and a goat anti-rabbit IgG (H + L) secondary antibody (Life Technologies).

### Statistical analysis

Data were statistically analyzed with a two-tailed unpaired Student’s *t* test by using GraphPad Prism 6 software. A *P* value of < 0.05 was considered to be statistically significant.

## Electronic supplementary material

Below is the link to the electronic supplementary material.

Supplementary material 1 (PDF 1254 kb)
