# Supplementary material for: TRIM35 mediates protection against influenza infection by activating TRAF3 and degrading viral PB2
Source: Protein Cell. 2020 Jun 19;11(12):894–914. doi: 10.1007/s13238-020-00734-6 (PMC7719147; doi:10.1007/s13238-020-00734-6)
Supplement: Supplementary file 1 — Supplementary material 1 (PDF 1254 kb) [file 13238_2020_734_MOESM1_ESM.pdf]

Table S1. Sequence analysis of residue 736 in the PB2 protein of IAV strains.

Total number of IAV strains analyzed[a]	Number and percentage of IAV strains with the indicated residue at position 736 of PB2		
	Residue	Number	Percentage (%)
59520	K	59508	99.98
	R	8	0.013
	unknown	4	0.007

[a]Sequences were derived from the Influenza Research Database, and were analyzed by using the ‘Analyze & Visualize’ module.

Table S2. SiRNAs used for gene silencing.

siRNA	Sequences (5'-3')
human *TRIM35*-sense	5'-GCAGGAGUUUGAUAAGCUUTT-3'
human *TRIM35*-antisense	5'-AAGCUUAUCAAACUCCUGCTT-3'
mouse *Trim35*-sense	5'-CCAACCACACGCUCAACAATT-3'
mouse *Trim35*-antisense	5'-UUGUUGAGCGUGUGGUUGGTT-3'
Scrambled-sense	5'-UUCUCCGAACGUGUCACGUTT-3'
Scrambled-antisense	5'-ACGUGACACGUU CGGAGAATT-3'

Table S3. Primers used for RNA quantification.

Primers	Sequences (5'-3')
hIFNB1-F	ATGACCAACAAGTGTCTCCTCC
hIFNB1-R	GCTCATGGAAAGAGCTGTAGTG
hIL6-F	CCTTCCAAAAATGGCAGAAA
hIL6-R	TCGATGCTTCCCTTATCACC
hISG56-F	GCCTTGCTGAAGTGTGGAGGAA
hISG56-R	ATCCAGGCGATAGGCAGAGATC
hTNFα-F	GCCGCATCGCCGTCTCCTAC
hTNFα-R	CCTCAGCCCCCTCTGGGGTC
hACTIN-F	CCTTCCTGGGCATGGAGTCCTG
hACTIN-R	GGAGCAATGATCTTGATCTTC
hTRIM35-F	CTTGAGAGTGGAGGAGCAGG
hTRIM35-R	GCGTTTTCGGCTCTTGTGTT
mIfnb1-F	CCCTATGGAGATGACGGAGA
mIfnb1-R	CTGTCTGCTGGTGGAGTTCA
mIl6-F	CTGCAAGAGACTTCCATCCAG
mIl6-R	AGTGGTATAGACAGGTCTGTTGG
mIsg56-F	TGCGATCCACAGTGAACAAC
mIsg56-R	ACTTCCGGGAAATCGATGAG
mTnfα-F	CAGGCGGTGCCTATGTCTC
mTnfα-R	CGATCACCCCGAAGTTCAGTAG
mActin-F	CCACACCCGCCACCAGTTCG
mActin-R	TACAGCCCGGGGAGCATCGT
mTrim35-F	CAAGGACACTGCGCAAGACTTC
mTrim35-R	TGCTTGGCAATGGCCTCATA

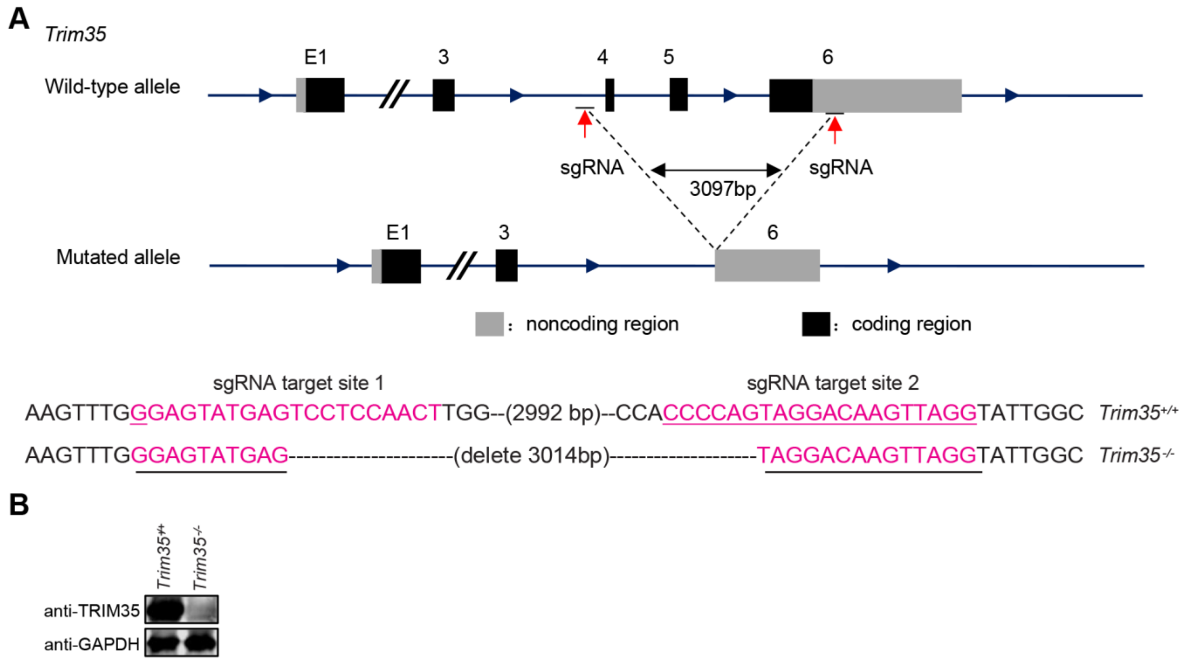

Figure S1. Generation of *Trim35*$^{-/-}$ mice by CRISPR/Cas9-mediated gene knockout. A) Schematic illustration of the strategy used to generate *Trim35*$^{-/-}$ mice. Two sgRNAs were designed to delete exons 4 to 6 of the *Trim35* gene. **B**) IB analysis of lung extracts of *Trim35*$^{+/+}$ and *Trim35*$^{-/-}$ mice.

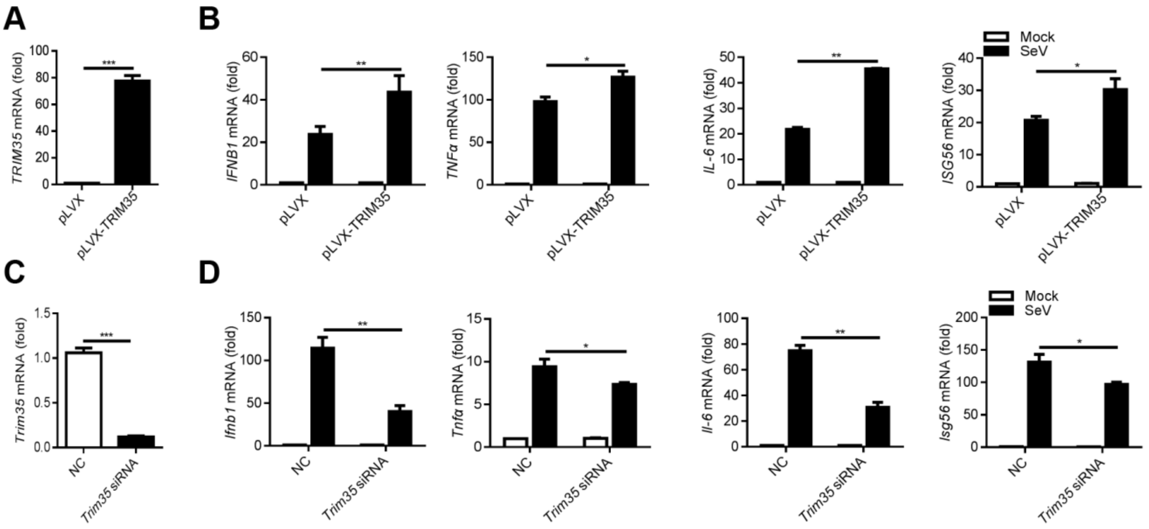

Figure S2. TRIM35 positively regulates the expression of IFN-β signaling pathway genes in THP-1 and RAW264.7 cells. **A**) Establishment of a stable lentiviral-mediated THP-1 cell line overexpressing TRIM35, confirmed by RT-qPCR. **B**) RT-qPCR analysis of the mRNA levels of the indicated genes in TRIM35-overexpressing or control THP-1 cells from **A**) that were left uninfected or were infected with SeV for 12 h. **C**) RT-qPCR analysis to assess the knockdown of TRIM35 in RAW264.7 cells transfected with *Trim35*-specific or scrambled siRNA for 36 h. **D**) RT-qPCR analysis of the mRNA levels of the indicated genes in siRNA-treated RAW264.7 cells from **C**) that were left uninfected or were infected with SeV for 12 h. Data are representative of at least three independent experiments. Means ± SD are shown in **A**)-**D**) (n=3). Two-tailed unpaired t-test was used for the statistical analysis, *$P < 0.05$, **$P < 0.01$, ***$P < 0.001$.

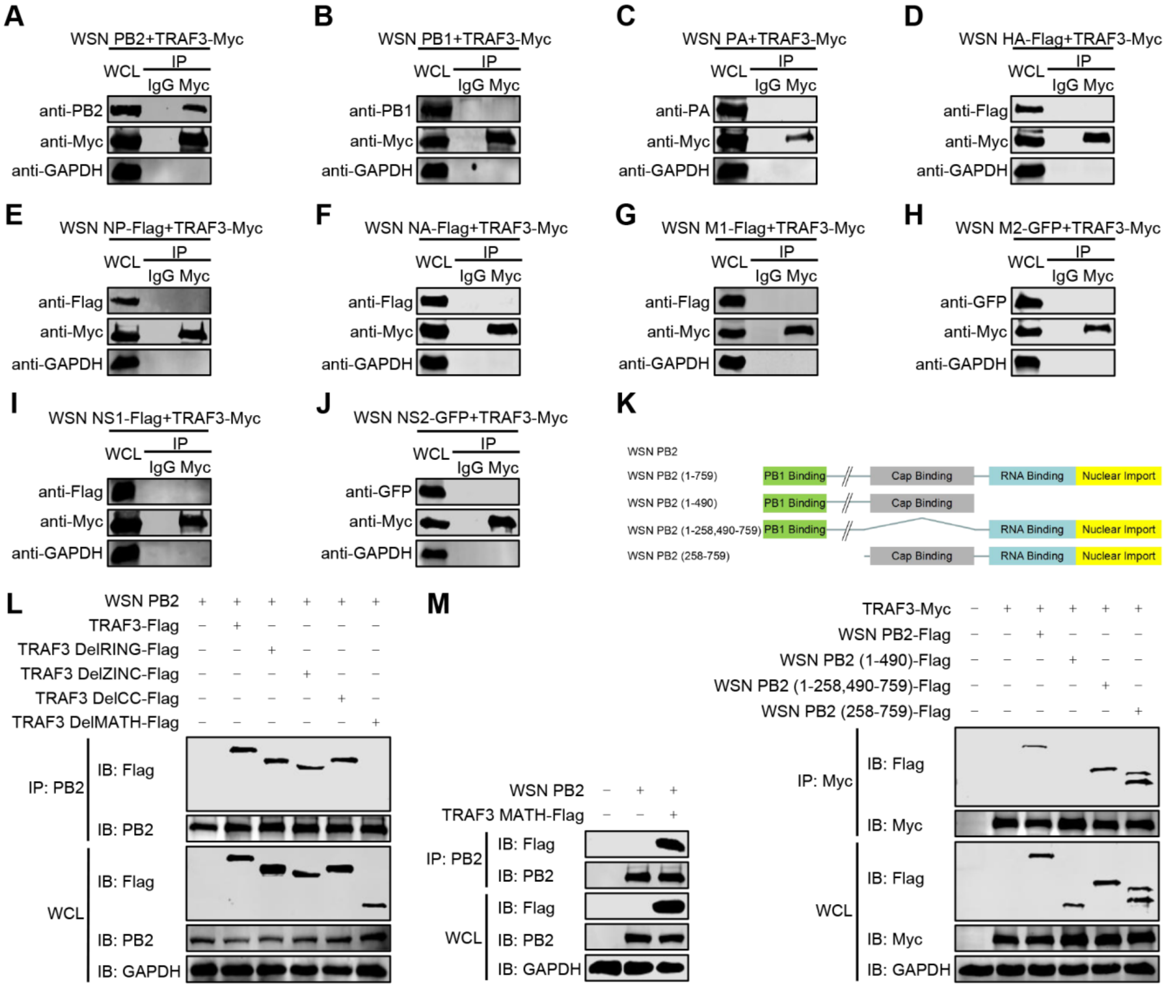

Figure S3. IAV PB2 interacts with TRAF3. A)-J) Co-IP and IB analysis of HEK293T cells expressing TRAF3-Myc and WSN (H1N1) PB2 **A**), PB1 **B**), PA **C**), HA-Flag **D**), NP-Flag **E**), NA-Flag **F**), M1-Flag **G**), M2-GFP **H**), NS1-Flag **I**), or NS2-GFP **J**). **K**) Binding of Flag-tagged WSN (H1N1) PB2 or its truncation mutants with TRAF3-Myc in transiently transfected HEK293T cells, as determined by co-IP and IB analysis. WSN (H1N1) PB2 domains and truncation mutants were shown on top. **L**) Binding of Flag-tagged TRAF3 or its truncation mutants with WSN (H1N1) PB2 in transiently transfected HEK293T cells, as determined by co-IP and IB analysis. **M**) Binding of Flag-tagged TRAF3 MATH domain with WSN (H1N1) PB2 in transiently transfected HEK293T cells, as determined by co-IP and IB analysis. Data are representative of at least three independent experiments.

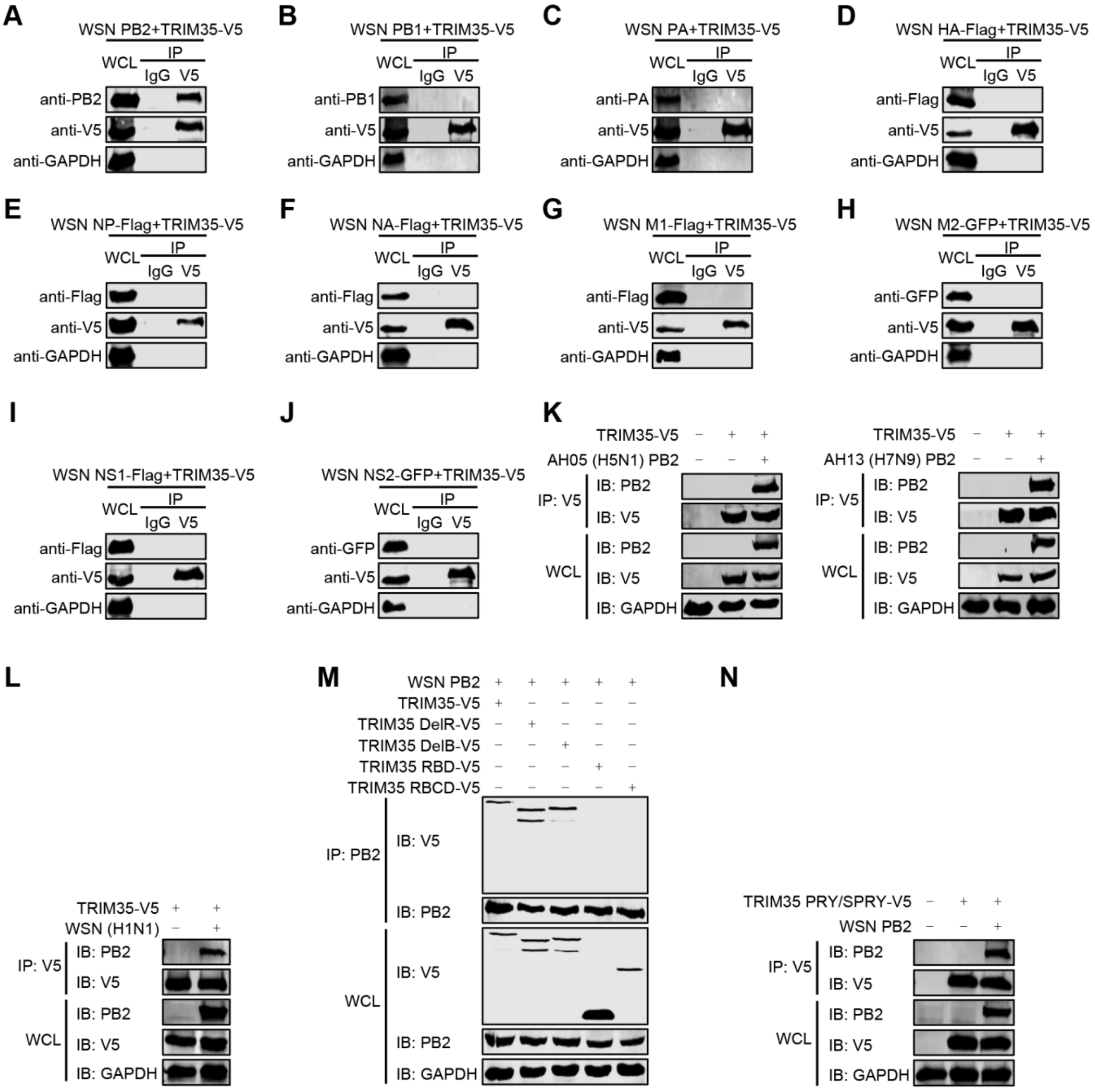

Figure S4. TRIM35 interacts with IAV PB2. **A**)-**J**) Co-IP and IB analysis of HEK293T cells expressing TRIM35-V5 and WSN (H1N1) PB2 **A**), PB1 **B**), PA **C**), HA-Flag **D**), NP-Flag **E**), NA-Flag **F**), M1-Flag **G**), M2-GFP **H**), NS1-Flag **I**), or NS2-GFP **J**). **K**) Co-IP and IB analysis of HEK293T cells expressing TRIM35-V5 and AH05 (H5N1) PB2 or AH13 (H7N9) PB2. **L**) Co-IP and IB analysis of HEK293T cells transfected with plasmid expressing TRIM35-V5 for 36 h, followed by infection with WSN (H1N1) virus for 12 h. **M**) Binding of V5-tagged TRIM35 or its truncation mutants with WSN (H1N1) PB2 in transiently transfected HEK293T cells, as determined by co-IP and IB analysis. **N**) Binding of V5-tagged TRIM35 PRY/SPRY domain with WSN (H1N1) PB2 in transiently transfected HEK293T cells, as determined by co-IP and IB analysis. Data are representative of at least three independent experiments.

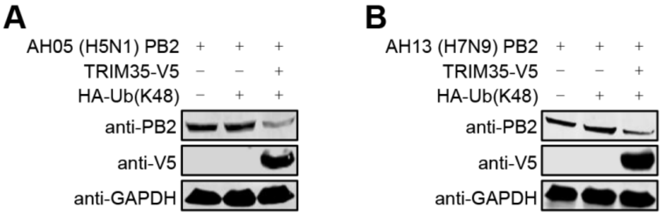

Figure S5. TRIM35-mediated K48-linked polyubiquitination leads to degradation of the PB2 protein of different IAVs. A) **and B**) IB analysis of HEK293T cells transfected for 36 h with plasmids expressing AH05 (H5N1) PB2 **A**) or AH13 (H7N9) PB2 **B**), with or without TRIM35-V5 and HA-ubiquitin (K48). Data are representative of at least three independent experiments.
